# More Than Eggs – Relationship Between Productivity and Learning in Laying Hens

**DOI:** 10.3389/fpsyg.2018.02000

**Published:** 2018-10-26

**Authors:** Anissa Dudde, E. Tobias Krause, Lindsay R. Matthews, Lars Schrader

**Affiliations:** ^1^Institute of Animal Welfare and Animal Husbandry, Friedrich-Loeffler-Institut, Celle, Germany; ^2^Department of Animal Behaviour, Bielefeld University, Bielefeld, Germany; ^3^School of Psychology, The University of Auckland, Auckland, New Zealand; ^4^Lindsay Matthews Research International, Hamilton, New Zealand

**Keywords:** domestication, selection, cognition, discrimination learning, poultry, laying hens, layer, extinction

## Abstract

The intense selection of chickens for production traits, such as egg laying, is thought to cause undesirable side effects and changes in behavior. Trade-offs resulting from energy expenditure in productivity may influence other traits: in order to sustain energetic costs for high egg production, energy expenditure may be redirected away from specific behavioral traits. For example, such energetic trade-offs may change the hens’ cognitive abilities. Therefore, we hypothesized highly productive laying hens to show reduced learning performance in comparison to moderate productive lines. We examined the learning ability of four chicken lines that differed in laying performance (200 versus 300 eggs/year) and phylogenetic origin (brown/white layer; respectively, within performance). In total 61 hens were tested in semi-automated Skinner boxes in a three-phase learning paradigm (initial learning, reversal learning, extinction). To measure the hens’ learning performance within each phase, we compared the number of active decisions needed to fulfill a learning criteria (80% correct choices for learning, 70% no responses at extinction) using linear models. Differences between the proportions of hens per line that reached criterion on each phase of the learning tasks were analyzed by using a Kaplan–Meier (KM) survival analysis. A greater proportion of high productive hens achieved the learning criteria on each phase compared to less productive hens (Chi^2^_3_ = 8.25, *p* = 0.041). Furthermore, high productive hens accomplished the learning criteria after fewer active decisions in the initial phase (*p* = 0.012) and in extinction (*p* = 0.004) compared to the less selected lines. Phylogenetic origin was associated with differences in learning in extinction. Our results contradict our hypothesis and indicate that the selection for productivity traits has led to changes in learning behavior and the high productive laying hens possessed a better learning strategy compared to moderate productive hens in a feeding-rewarding context. This better performance may be a response to constraints resulting from high selection as it may enable these hens to efficiently acquire additional energy resources. Underlying mechanisms for this may be directly related to differences in neuronal structure or indirectly to foraging strategies and changes in personality traits such as fearfulness and sociality.

## Introduction

Domestication, a process whereby an animal is shaped genetically and phenotypically while living under human supervision (Price, 1999; Jensen, 2006), led to immense but similarly directed phenotypical and behavioral changes of animals, the so-called domesticated phenotype (Jensen, 2014). More recently, intense selection for productivity traits of domesticated livestock animals has led to additional phenotypic and behavioral changes, which are considered to have potential negative effects on animal welfare (Rauw et al., 1998).

The modern chicken, *Gallus gallus domesticus*, is a good example for this process, as its behavior has been influenced and shaped by domestication and later on by selection for productivity traits (Hale, 1962; Price, 1998; Schütz and Jensen, 2001). Their domestication was not a linear process and occurred several times at different places in the world (Wood-Gush, 1959; Lyimo et al., 2014; Woldekiros and D’Andrea, 2017), leading to phylogenetic variation in layer chickens with strain-specific features (de Haas et al., 2013; Lyimo et al., 2014). Still, all domesticated chickens share, to a certain degree, similar phenotypical and behavioral modifications in comparison to the ancestral red jungle fowl (Jensen, 2006).

Modern layer breeds still do exhibit mostly the same behavior repertoire as the red jungle fowl, but with changed frequencies and/or intensities (Hale, 1962; Price, 1998; Schütz and Jensen, 2001; Jensen, 2006). Further, selection of domestic hens for increased productivity traits, such as egg laying, may have led to additional modification of specific behavioral traits, like reduced aggression or sociality (Schütz and Jensen, 2001; Schütz et al., 2001; Väisänen and Jensen, 2003; Lindqvist and Jensen, 2009).

Chickens are known to possess a complex behavioral repertoire, including sophisticated cognitive abilities (Krause et al., 2006; Nicol, 2015; Marino, 2017; Garnham and Løvlie, 2018). Like other animals, they use learning as a key mechanism to adapt to their physical and social environment (Jensen, 2006). This appears first directly after hatching with remarkable filial imprinting (Bolhuis, 1991) and continues throughout life, for example, in foraging contexts, where chickens learn to orientate, (re)locate specific food resources, or can be trained in artificial situations to use operant feeders (Nicol, 2015). Understanding the cognitive abilities of chickens and other livestock animals can have crucial impact on their husbandry and production and thus on their welfare (Nicol, 1996; Abeyesinghe et al., 2005; Smith and Johnson, 2012). As humans tend to expect animals with greater cognitive similarities to humans to be more likely to suffer (Serpell, 2004; Smith and Johnson, 2012), which is, from a scientific perspective, not reasonable (Dawkins, 2001). However, as domestication and selection have influenced particular behavioral traits, the contingent question arises, whether the cognitive abilities of domesticated and selected animals have been influenced and altered as well. In chickens, Lindqvist and Jensen (2009) have shown that the domesticated, high selected White Leghorn chickens (males and females) perform worse in a spatial learning task, compared to the ancestral red jungle fowl. However, it is difficult to say, whether the observed differences were related to the phylogenetic origin of the chickens, effects of domestication or effects of selection for productivity traits (see also Schütz et al., 2001).

An underlying mechanism, which could cause behavioral changes as reduced learning abilities in highly selected animals could be productivity-induced trade-offs, which are also predicted by the resource allocation theory (Beilharz et al., 1993; Mignon-Grasteau et al., 2005). Evolutionary adaptation should have resulted in an optimal energy allocation between self-preservation and reproductive processes in order to maximize evolutionary fitness in wild animals, like the red jungle fowl. Schütz and Jensen (2001) have hypothesized that modern layers, in contrast, may have shifted more of their energy resources toward reproduction, e.g., increased egg yield, which could lead to trade-offs represented in the behavior of the chickens.

It seems to be reasonable to assume that trade-offs might have occurred, as egg productivity increased over the last decades in layer hybrids by about 1% per year, which equates to about two additional eggs per year (Flock and Heil, 2002; McKay, 2008). Annual egg production of laying hens was at about 150 eggs in the 1940s (Klauder, 1948), whereas today, annual egg production is about 300. Layers of both white and brown lines produce more than 300 eggs/year with a very high feed conversion efficiency (Lieboldt et al., 2015a). Neuronal processes are metabolically particular costly (Brady et al., 2011; Bullmore and Sporns, 2012; Kuzawa et al., 2014), which led us to the assumption that trade-offs in cognitive skills are likely to have taken place in response to the increased egg yield (Van der Waaij, 2004; Mirkena et al., 2010).

Thus, our aim in this study was to determine whether intensive selection of domesticated layer lines for high egg yields has altered their cognitive skills. Hence, we focus, as a proxy for these skills, on the learning abilities and the flexibility in learning. We tested hens of four domesticated chicken lines in an operant conditioning task, consisting of (i) discrimination learning, (ii) a reversal learning, followed by (iii) an extinction procedure.

The four laying lines we tested, varied, using a crossed design, in their level of egg yield and their phylogenetic origin (white versus brown shell layers). Thus, two laying lines were high productive: WLA (originating from White Leghorn, 325 eggs/year) and BLA (originating from Rode Island, 310 eggs/year). The two others were moderate productive lines: R11 (originating from White Leghorn, 200 eggs/year) and L68 (originating from New Hampshire, 205 eggs/year). Growth rates, feed conversion rates, productivity, and other relevant physiological characteristics of this lines have been described in great detail in previous studies (Granevitze et al., 2009; Lieboldt et al., 2015a,b, 2016; Polasky et al., 2016; Höhne et al., 2017). For example, is the daily feed intake per mass of hens 68.73 g food/kg for WLA, 62.61 g/kg for BLA and lower for the moderate productive lines with 51.98 g/kg for L68 and 58.37 g/kg for R11 (Lieboldt et al., 2015a). According to those performance data, both high productive lines are similar to each other, as well as both moderate productive lines. A specific phylogenetic characteristic is that both brown layer lines, i.e., BLA and L68, are heavier than both white layer lines (Lieboldt et al., 2015a).

We expected that if cognitive abilities of the hens have been altered in response to the selection for productivity, the two high productive lines should behave similarly regardless of their different phylogenetic background and that the same should be true for the two moderate productive lines. Furthermore, we assume resource trade-offs to appear and therefore that hens of high productive laying lines would possess reduced learning performances, indicated by reduced learning abilities and slower flexibility, compared to hens with a lower level of egg yield.

## Materials and Methods

### Animals and Housing

We used hens of four purebred layer lines (*Gallus gallus domesticus*) differing in two dimensions in a crossed two by two design: (i) egg laying performance: high annual egg laying rate of approximately 310 eggs/year and moderate annual egg laying rate of approximately 200 eggs/year and (ii) phylogenetic origin: white layers that lay eggs with white shells and brown layers (Lieboldt et al., 2015b). The two lines with high annual egg performance are the WLA (high egg laying performance and white layer) and the BLA (high egg laying performance and brown layer). Both are originating from Lohmann Tierzucht GmbH. The two lines with moderate egg laying performance are the R11 (moderate egg laying performance and white layer) and L68 (moderate egg laying performance and brown layer). These lines originated from resource populations at the Institute of Farm Animal Genetics, Friedrich-Loeffler-Institut (FLI), Mariensee, Germany. R11 has been kept at the FLI since the 1960s (Hartmann, 1987), and L68 was bred in the 1970s (VEG Vogelsang, Lieboldt et al., 2015b). The two white layer lines, WLA and R11, are of White Leghorn origin and cluster phylogenetically close together (Granevitze et al., 2009; Lyimo et al., 2014), but they are genetically distant from the two brown layers. Also both brown layer lines, BLA and L68, cluster genetically close together (Lyimo et al., 2014). BLA originates from Rhode Island, whereas L68 originates from New Hampshire (model described in Granevitze et al., 2009; Lieboldt et al., 2015a,b, 2016; Polasky et al., 2016; Höhne et al., 2017; Krause and Schrader, 2018). Chickens from all four lines were incubated and hatched simultaneously at the Institute of Animal Welfare and Animal Husbandry, FLI, Celle, Germany. At hatching, each chick was equipped with an individually numbered wing-tag for identification. All chicks were raised together and under identical conditions until the 16th week of age. Thereafter, the hens were kept in four adjacent compartments in a stable, separated by line, with 4 m^2^ floor area each. Here, the hens had access to group nests, perches, litter, pecking blocks and an additional sand tank for dustbathing. In their home compartments, the hens had *ad libitum* access to water and standard commercial layer food. The light–dark cycle was set to 14 L:10 D. For this experiment, we used a total of 61 hens (BLA: *n* = 17, L68: *n* = 18, WLA: *n* = 13, and R11: *n* = 13), that were 45 weeks old at the beginning of the experiment, thus in the laying period.

### Experimental Setup

**FIGURE 1:**
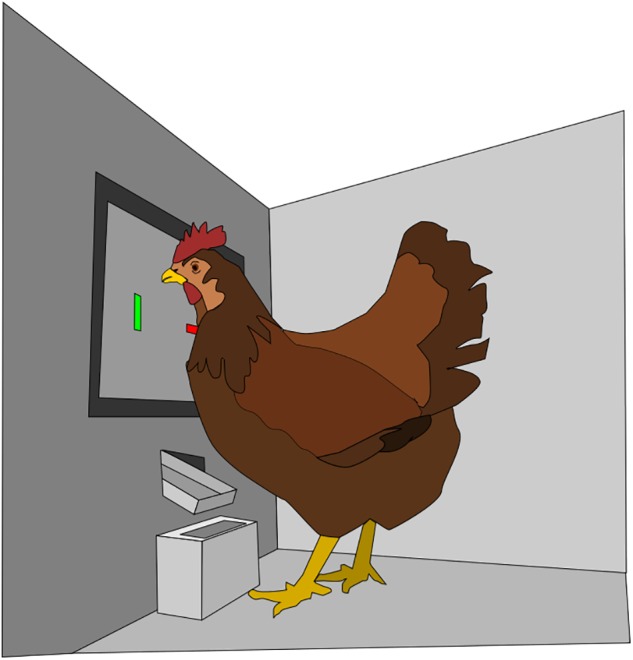
Schematic illustration of the test box (width, depth, height: 55 cm × 46.5 cm × 66 cm). The black touchscreen (height × wide: 19 cm × 25 cm, diagonal: 31 cm) is on the back wall, displaying two different stimuli, the colored bars. Wheat grain rewards are delivered to a food trough (height × wide × depth: 1.5 cm × 4 cm × 8 cm) below the screen. The walls are made out of clear plexiglass.

The whole experiment, i.e., habituation, screen training, and the three learning phases, were carried out in four identical custom-built test-boxes (Figure 1), located in an adjacent room to the home-compartments of the hens. Each box consisted of plexiglass walls (width, depth, height: 55 cm × 46.5 cm × 66 cm) with a TFT monitor on one side (model DT-121-A from Distronic (Distronic, Hochheim/Main, Germany)). The display of this monitor (12.1 in (height × wide: 19 cm × 25 cm,) was a SVGA 600 × 800 pixel model (LB121S02-TD01 from Philips (Philips Deutschland GmbH, Hamburg, Germany)). Over the monitor laid a frame (IR Touch-kit 121.-A301, Citron GmbH, Augsburg, Germany) at a distance of 1.0 cm. This frame created a mesh of infrared light beams across the monitor in order to recognize pecks. When a beam was broken, e.g., when a chicken beak pecked against the monitor, the position was recorded by *x*- and *y*-coordinates. Underneath this set up of screen and frame, a foldable food trough (height × wide × depth: 1.5 cm × 4 cm × 8 cm) was placed (see Figure 1). To reward a hen, this trough could be filled with wheat grains (approximately 22 wheat grains, e.g., 2 g, per turn) by a computer controlled disperser (model craft, RB350-600-0A101R, Conrad Electronics, Hirschau, Germany). The time a hen was enabled to feed, in case of a reward, could be controlled and was 5 s in the screen training and the three learning levels. Thereafter, the trough was automatically cleared. The amount of the food reward was higher than the hens could eat during that time. The wheat grains were stored in a container outside the box and not visible to the tested hens. When a food reward was delivered to the hen, a small white LED light close to the through turned on as well. Loudspeakers (Logitech PC stereo Z120 1.2W, Logitech Europe S.A., Lausanne, Switzerland) were placed above the monitor in the corners of the test-boxes, playing acoustic sound (*Windows Default*) when the screen was touched. A custom-made computer program, written in C++ (Microsoft Visual Studio, 2010) controlled the complete electronic set up of the box, e.g., sound, light, reward delivery, touchscreen, and monitor. For additional observation of the hens, a video camera was installed above each learning test-box. The hens were not able to see each other from inside the box.

### Training, Testing, and Learning Criteria

In this experiment, the hens were observed in successive habituation, screen training, and learning phases, consisting of differential learning, reversal learning and extinction (see Table 1). While training and testing, the hens could obtain wheat grain rewards and had always *ad libitum* access to food and water in their home compartments. Thus, the hens were not food restricted. The protocol of training this operant conditioning task was adapted after Mar et al. (2013). For all experimental phases, the hens were individually taken from their home compartments and gently placed in to the test-boxes. Each day, 4 days a week, the hens stayed in the test box for one session to a maximum of 20 min. The time in the box, separated from the conspecifics, was slowly increased during the habituation phase (see Table 1). However, if a hen participated in the experiment and made quick decisions, it could decrease its time in the box, since each hen was asked to make 20 decisions per session (equal 20 trials, see Supplementary Figure S3). Alternatively, the time in the box ended after the 20 min.

**Table 1:** Phases of the experiment and their specific characteristic.

	Level	Time	Stimulus	Task/reward for	Learning criteria
Habituation	0	One session	None	Stay 5 min in the box and eat wheat grain *ad libitum*	None
		One session	None	Stay 10 min in the box and eat wheat grain *ad libitum*	None
		One session	None	stay 10 min in the box and eat wheat grain *ad libitum*, turning on and off of reward delivery system	None
		One session	None	Stay 15 min in the box and eat wheat grain only when reward system turns on, time to eat 20 s	None
		One session	None	Stay 15 min in the box and eat wheat grain only when reward system turns on, time to eat 5 s	None
Screen training	1	Individual	Circle	Peck on circle or no peck on circle within 30 s – rewarded	80% Correct
	2	Individual	Circle	Peck three times on circle – rewarded	80% Correct
	3	Individual	Circle	Peck five times on circle – rewarded	80% Correct
Discrimination	4	Individual	Bars	Peck five times on correct symbol – rewarded	80% correct
Reversal	5	Individual	Bars	Peck five times on correct symbol – rewarded	80% Correct
Extinction	6	Individual	Bars	No response, not rewarded	70% Correct

*The amount of sessions a hen needed for screen training and the learning phases depended on the individual learning behavior.*

If a hen did not succeed to finish one of the screen training levels or one of the three learning phases within 20 sessions, it was excluded from the further experiment. To successfully finish one of those phases, except the extinction, a hen needed 80% correct decisions out of at least 10 decisions. This learning criteria differs from 50% chance level and is in accordance to other learning studies (e.g., Garner et al., 2006; Nawroth et al., 2014; Brust and Guenther, 2015). To successfully finish the extinction, the hen needed to demonstrate no responses in 70% of at least 10 trials.

### Stimuli

The chosen stimuli, a gray circle (diameter: 2 cm, color in RGB values: R = 224, G = 224, B = 224) for screen training and a green bar (high × length: 10 mm × 40 mm, color in RGB values: R = 20, G = 184, B = 29) and a red bar (high × length: 10 mm × 40 mm, color in RGB values: R = 237, G = 28, B = 36, see Supplementary Figures S1, S2) for the three different learning level, where presented on a black screen and were all detectable for the hens visual physiology (Osorio et al., 1999).

### Habituation and Screen Training

The habituation phase was subdivided into five sessions (Table 1). Throughout this, the hens were slowly trained to stay calm and separated in the test box (Figure 1) and to find the food rewards in the trough. For this habituation phase, the test hen was placed in the box on the first day for 5 min with *ad libitum* access to wheat grains. During the 4 following days, the hen’s time in the box increased while the access to the food reward decreased. On the fifth day of habituation, a hen stayed 15 min in the box and wheat grains were given though the delivery system with 5 s feeding time (Table 1).

After that, the hens continued with the screen training phase, which amount of sessions depended on the individual participation of the hens. While screen training, the hens learned to use the screen in combination with the food reward delivery system. As a stimulus for this, we used gray circle on black background (2 cm diameter, see Supplementary Figure S1) presented at a randomized position on the touchscreen. The screen training was again subdivided in tree level during which the hens were successively trained to peck five times on the circle in order to receive a reward (see Table 1). When a hen finished the habituation sessions and screen training level successfully, it was allowed to continue with the learning tasks.

### Learning Tasks

#### Phase 1: Discrimination Learning

For the discrimination learning, the hens needed to learn to differentiate between two simultaneously shown colored bars, red and green (see Supplementary Material), independent of the bars orientation. It was randomly selected, whether a hen learned that red or green was the rewarded color. Furthermore, the side of the screen on which the rewarded bar appeared was randomized, to avoid side preferences (de Haas et al., 2017a,b). Pecking on the black screen was neither rewarded nor counted as a wrong decision. If a hen made a correct decision, thus pecking on the correct bar, it was rewarded and allowed to feed on wheat grains that were provided in the trough for 5 s before the next trial appeared. Therefore, a black screen was shown for 20 s (inter-component time) and after that, the two colored bars appeared again with a randomized position (left or right) and orientation (horizontal or vertical). If a hen made a wrong decision, no reward was given, and a black screen appeared for 5 s, followed again by 20 s of inter-component time. After that, the previous shown bars appeared at the same position again (correction trial, see Supplementary Figure S3). Hens solved the differential learning when they made 80% correct decisions of at least 10 decisions.

#### Phase 2: Reversal Learning

When a hen entered the reversal learning phase, the initial unrewarded color was rewarded and the initial rewarded color was unrewarded. Everything else, e.g., inter-component time and feeding time of the reward remained the same. The reversal learning was successfully finished after 80% correct decisions of at least ten decisions. This form of learning provides two simultaneous learning tasks, and the previously learned association needs to be deleted while a new association needs to be learned (Coppens et al., 2010; Brust et al., 2014; Zidar et al., 2017).

#### Phase 3: Extinction

In the extinction phase, no food reward was provided and the extinction criteria was reached, when a hen did not respond to any of the symbols on the screen in 70% of a least 10 trials. If a hen did not peck, the symbols vanished after 20 s, followed by an inter-component time of 20 s. If a hen pecked on one of the symbols on the touchscreen, the black screen appeared for an inter-component time of 20 s.

### Data Analysis

In order to compare the proportion of hens per line that achieved criterion on each phase of the screen training and learning task, we counted the number of hens per line, which were still participating in accordance to the above-mentioned criteria in the test at each phase. These numbers were analyzed them by using a Kaplan–Meier (KM) survival analysis (for criteria, see section “Materials and Methods”).

To compare the learning performance of the hens, we analyzed the sum of their active decisions needed, to fulfill the learning criteria. The active decisions are the amount of correct and wrong decisions, whereby inactive trials with no decisions are not implicated. Throughout this, we aimed to correct for confounding motivation since a “no decision” does not reveal actual information about the learning process itself and might be likely influenced by other factors. The residuals of the average number of active decisions per phase were normal distributed and homogeneity of the variances was given; therefore, we analyzed the data with linear models (LMs). The two by two designed LMs consisted of the factors: phylogeny (two levels: white and brown layers) and productivity (two levels: high and moderate productive hens) and their interaction. Non-significant factors were not excluded from the model according to the recommendation of Forstmeier and Schielzeth (2011).

The data processing was performed using a custom – written Matlab (Matlab and Statistics Toolbox Release, 2017) script to summarize the data per training and learning level. The data per phase for the LMs were statistically analyzed using R 3.3.1, R Core Team (R Core Team, 2016) and Statistica 13 (Statistica, 2015) for the survival analysis.

### Ethical Note

This study was approved by the German Lower Saxony State Office for Consumer Protection and Food Safety (LaVes) (# 33.19-42502-04-15/5054) and in accordance with German regulations on Animal Welfare.

## Results

### Success in Participating Throughout the Test

**FIGURE 2:**
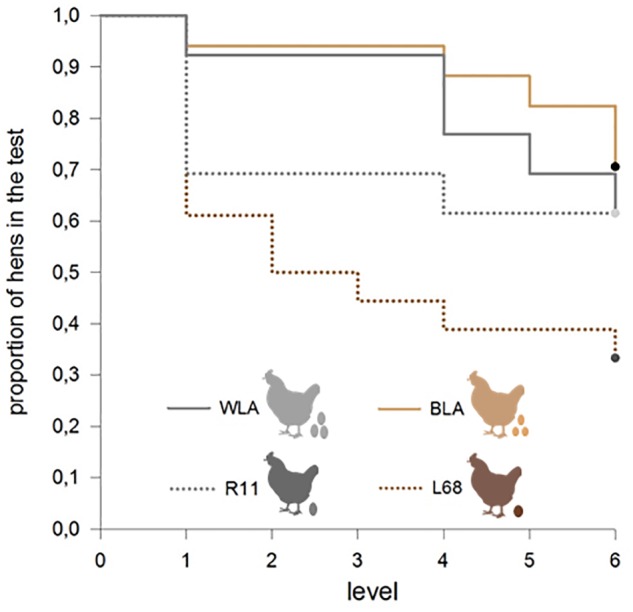
Proportion of hens that successfully pass the learning tasks of each level. A greater proportion of high productive hens, WLA and BLA, achieved the learning criteria at the screen training and learning level, compared to moderate productive hens, R11 and L68 (Chi^2^_3_ = 8.25, *p* = 0.041).

The proportion of successful participation in the experiment differed between the four tested lines. A greater proportion of high productive hens, WLA and BLA, achieved the learning criteria on the screen training and learning levels, compared to moderate productive hens, R11 and L68 (Chi^2^_3_ = 8.25, *p* = 0.041, see Figure 2).

### Learning Performance

**FIGURE 3:**
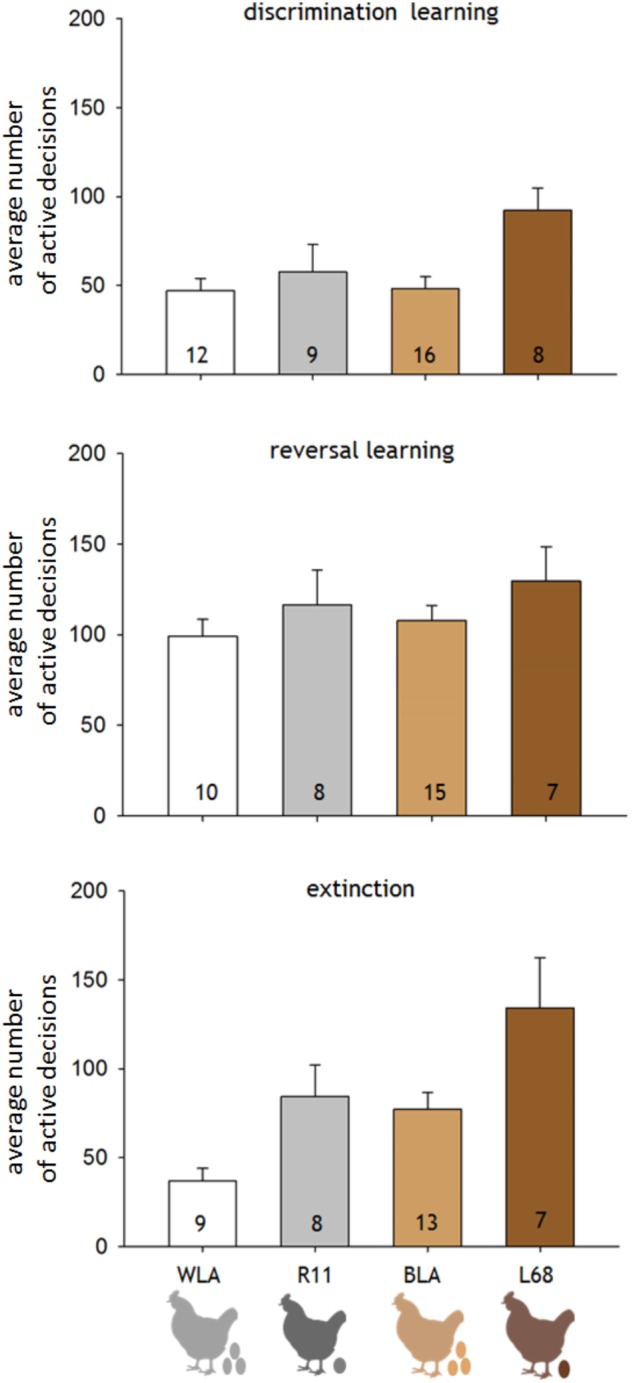
Averaged number of active decisions (±SD) needed by hens of the different lines, WLA, R11, BLA, and L68, to fulfill the learning criteria of each phase. The number within each column represents the number of hens, which participated in that phase (*n*). Discrimination learning was significant affected by productivity (LM: factor productivity: *F*_1,41_ = 69.63, *p* = 0.011; factor phylogeny: *F*_1,41_ = 2.023, *p* = 1.63; interaction productivity^∗^phylogeny: *F*_1,41_ = 2.792, *p* = 0.102). Whereas reversal learning was more difficult for all hens, independent of their phylogenetic background or productivity level (LM: factor productivity: *F*_1,36_ = 1.924, *p* = 0.174; factor phylogeny: *F*_1,36_ = 0.641, *p* = 0.423; interaction productivity^∗^phylogeny: *F*_1,36_ = 0.028, *p* = 0.867). The average active decisions needed for the extinction level were significantly affected by phylogenetic origin and productivity (LM: factor productivity: *F*_1,33_ = 9.543, *p* = 0.004; factor phylogeny: *F*_1,33_ = 8.588, *p* = 0.006; interaction productivity^∗^phylogeny: *F*_1,33_ = 0.09, *p* = 0.766).

The differential learning performance was significantly affected by the different productivity levels of the hens, but not by differences in the phylogeny (LM: factor productivity: *F*_1,41_ = 69.63, *p* = 0.011; factor phylogeny: *F*_1,41_ = 2.023, *p* = 0.163; interaction productivity^∗^phylogeny: *F*_1,41_ = 2.792, *p* = 0.102; Figure 3). The extinction was significantly affected by productivity and phylogeny (LM: factor productivity: *F*_1,33_ = 9.543, *p* = 0.004; factor phylogeny: *F*_1,33_ = 8.588, *p* = 0.006; interaction productivity^∗^phylogeny: *F*_1,33_ = 0.09, *p* = 0.766; Figure 3). Thus, high productive hens, WLA and BLA, accomplished the learning criteria after fewer active decisions in the initial learning phase and the extinction, compared to the moderate productive lines, R11 and L68. Furthermore, the white hens, WLA and R11, needed less active decisions in the extinction compared to brown layers (Figure 3).

In the reversal learning, all hens needed a similar amount of active decisions to fulfill the learning criteria (LM: factor productivity: *F*_1,36_ = 1.924, *p* = 0.174; factor phylogeny: *F*_1,36_ = 0.641, *p* = 0.423; interaction productivity^∗^phylogeny: *F*_1,36_ = 0.028, *p* = 0.867; Figure 3).

## Discussion

We found that a higher proportion of high productive hens achieved the learning criteria on each of the learning tasks compared to the moderate productive hens. Furthermore, high productive hens accomplished the learning criteria after fewer active decisions in the initial learning and in extinction compared to the hens of moderate productive lines. Reversal learning requires most likely higher levels of flexibility in learning and was solved similarly by hens from both lines and productivity levels. Phylogenetic background was only associated with differences in learning ability in extinction; white layer hens performed better than the brown lines. Both the higher success of the high productive lines in the different phases and their lower number of active decisions required to fulfill the learning criteria in the initial discrimination learning and extinction in this experiment indicate that there is no trade-off between their cognitive abilities in favor of egg laying. Rather, these hens seem to possess a more efficient learning strategy. The fewer active decisions to learn imply a faster association of the visual-acoustic cue with the food reward. This is particularly interesting in extinction, where no food is available and any activity results in additional energy expenditure. Thus, our initial hypothesis, that high productive laying hens would be worse in learning, caused by energy trade-offs, cannot be proven in this experiment. The opposite seems to be the case but is still highlighting that the effect of the recent intense selection for high egg yields has altered behavior and cognitive skills of laying hens. Speculatively, the observed more efficient learning in the high productive hens may be a strategy to optimize energy intake. However, direct effects of this in high selected hens may be related to changes in the brain structure and indirectly to changes in food motivation or changes in personality traits.

A factor ensuring the hens to participate in the learning tasks is the food reward, while the value of that reward may vary between hens from the different lines. The high productive laying hens have been shown to possess a higher energy and nutrition demand than the moderate productive layers, due to higher egg lay production (Lieboldt et al., 2015a). It is likely that this has resulted in an increased food motivation. Such an increase of food motivation has been observed in meat chickens intensively selected for intensive body growth (Bokkers and Koene, 2002). Thus, a higher motivation to obtain the reward may have contributed to the observed differences in learning ability between the productivity lines. However, it is worth noticing that the hens in our study had *ad libitum* access to food in their home pens and no food deprivation prior to testing so motivational differences may be relatively minimized. In addition, by analyzing the active decision, we further reduced a possible impact of motivation. Nevertheless, it would be interesting to rerun the experiments with another reward system than food, e.g., social reinforces or enabling comfort behavior like dust bathing. In order to get further information on whether the actual egg laying directly affects the learning performance, it would be also interesting to test the males of each line. This would reveal further information on, whether the selection for egg productivity selectively targets the female’s genetical makeup.

Another reward-related influence on the results may be connected to differences in foraging strategies. Support for the idea that foraging strategies may have changed through selection is provided in the study by Schütz and Jensen (2001), who compared the behavior of high productive White leghorn layer hybrids with the red jungle fowl and the Swedish bantam chicken, a domesticated chicken line, which, however, is not selected for productivity. In their study, hens could either feed from an undiluted *ad libitum* food source or use a bowl where they needed to search and sort for food in wood-shavings (Schütz and Jensen, 2001). The high productive laying hens were more likely to eat from the undiluted food source, while the other two lines performed more foraging behavior and fed more often from the food source where searching and sorting was required. A similar underlying process might contribute to our findings, i.e., the higher number of active decisions needed to obtain a food reward shown by the moderate productive lines. These hens could use a more flexible foraging strategy, making them more likely to try the unrewarded stimulus, thereby requiring more active decisions to fulfill the learning criteria. Considering the moderate productive laying hen, as a slightly more native line, it can be argued that a flexible foraging strategy would be more effective in a natural environment, where resource patches are finite and variable. Higher flexibility provides the opportunity to learn about possible alternative food resources (Schütz and Jensen, 2001). High productive laying hens, arguably more adapted to domestic environments with secure food supplies can be more effective with a less flexible feeding strategy. Therefore, the high productive hens may have been more focused on exploiting one food source and may not even try other alternatives, once a successful strategy had been developed. To further investigate this idea, it would be also interesting to test other rewards, as mentioned above.

An important factor, which can influence the learning abilities of animals, is stress (Mendl, 1999). Stress can be caused by different factors, for example, by neophobia, general fearfulness or social stress, for e.g., caused by social isolation (Mendl et al., 1997; Mendl, 1999; Shaw and Schmelz, 2017), in which intensity an animal perceives or is affected by stress can also be related to its personality traits (de Haas et al., 2017a). Personality traits, in general, have been shown to influence learning in wild as well as in domesticated animals (Lansade and Simon, 2010; Guenther et al., 2014; de Haas et al., 2017a,b; Zidar et al., 2017). Furthermore, those traits have been described in fowl (Favati et al., 2015; Zidar et al., 2017; Dudde et al., 2018) and traits like sociality or fearfulness show some degree of heritability (Jensen, 2006; Garnham and Løvlie, 2018). Fearful hens, for example, may be poorer learners than less-fearful conspecifics (de Haas et al., 2017a,b). Further, Schütz et al. (2001) indicated that high productive laying hens may have a lower level of social motivation. Therefore, it is likely that in the here presented experiment, high productive lines may experience less social stress when separated from the flock for testing and therefore perform better in the given task. Such a result could be shown in goats, where individuals with a lower level of social motivation performed better in a visual discrimination task (Nawroth et al., 2017). In general, the here tested chicken lines could vary in their distribution of personality types (Dudde et al., 2018). Hence, in our learning task, phylogenetic background could have led to associated differences in the personality between the lines, which in turn, may have affected cognitive performance.

Nevertheless, it is possible that through the intensive selection process, the cognitive abilities in associative learning contexts of high productive hens have indeed improved. Underlying neurological mechanism that might have led to such an improved cognitive ability remains subject to speculation. Potentially, it could be related to adaptations of the neuronal structures, like neuronal density or lateralization, in high productive hens. Other studies have shown that brain structures can affect cognitive performance, e.g., the lateralization in chickens (Rogers et al., 2004; Daisley et al., 2010). The domestication process itself had a marked effect on the brain of domesticated animals, e.g., their brain size decreased (Kruska and Steffen, 2013). However, brain size itself does not correlate with cognitive function but more likely relates to changes in neuronal perception of acoustic or visual stimuli (Chittka and Niven, 2009). Therefore, it might be interesting to investigate from a behavioral perspective whether the sensitivity toward physical cues of high selected laying hens has altered. From a neurobiological perspective, it could be interesting to compare brain allometry, neuronal density, or lateralization of high selected laying hens in comparison to moderate productive lines.

## Conclusion

Taken together, our results demonstrate that laying hens have good cognitive abilities, as they can learn reasonably complex tasks. In contrast to our initial hypotheses, these abilities do not seem to be limited by resource trade-offs, resulting from high selected levels of egg lay capacity. Instead, it seems that the selection for productivity traits has led to changes in learning behavior and the high productive laying hens showed a better learning performance compared to moderate productive hens in a feeding-rewarding context. These higher levels of performance may be in response to constraints imposed by high selection pressure on productivity, resulting in more efficient strategies to gain additional energy, which may ameliorate the trade-offs from selection on high egg yields. Underlying mechanisms for this may be directly related to differences in neuronal structure or indirectly to foraging strategies and changes in personality traits such as fearfulness and sociality.

## Author Contributions

AD, EK, LM, and LS conceived and designed the study and wrote the manuscript. AD collected the data. AD and EK analyzed the data.

## Conflict of Interest Statement

The authors declare that the research was conducted in the absence of any commercial or financial relationships that could be construed as a potential conflict of interest.
