# Supplementary material for: More Than Eggs – Relationship Between Productivity and Learning in Laying Hens
Source: Front Psychol. 2018 Oct 26;9:2000. doi: 10.3389/fpsyg.2018.02000 (PMC6212530; doi:10.3389/fpsyg.2018.02000)
Supplement: Supplementary file 1 [file Data_Sheet_1.pdf]

More than eggs - Relationship between productivity and learning in laying hens

Anissa Dudde[1,2]*, E. Tobias Krause[1], Lindsay R. Matthews[3], Lars Schrader[1]

[1] Institute of Animal Welfare and Animal Husbandry, Friedrich-Loeffler-Institut, Dörnbergstr. 25/27, 29223 Celle, Germany

[2] Department of Animal Behaviour, University of Bielefeld, Konsequenz 45, 33615 Bielefeld, Germany

[3] University of Auckland, 22 Princes Street, Auckland, New Zealand and Lindsay Matthews Research International, 31 Rosebanks Drive, Hamilton 3283, New Zealand

*Corresponding author: anissa.dudde@fli.de

Supplement

Stimuli

The stimuli used in the experiment were a grey circle (diameter: 2 cm, color in RGB values: R = 224, G = 224, B = 224, see Fig. S1) for screen training. A green bar (high x length: 10 mm x 40 mm, color in RGB values: R = 20, G = 184, B = 29) and a red bar (high x length: 10 mm x 40 mm, color in RGB values R = 237, G = 28, B = 36 , see Fig. S2) were used for the three different learning level. All stimuli were represented on a black screen.

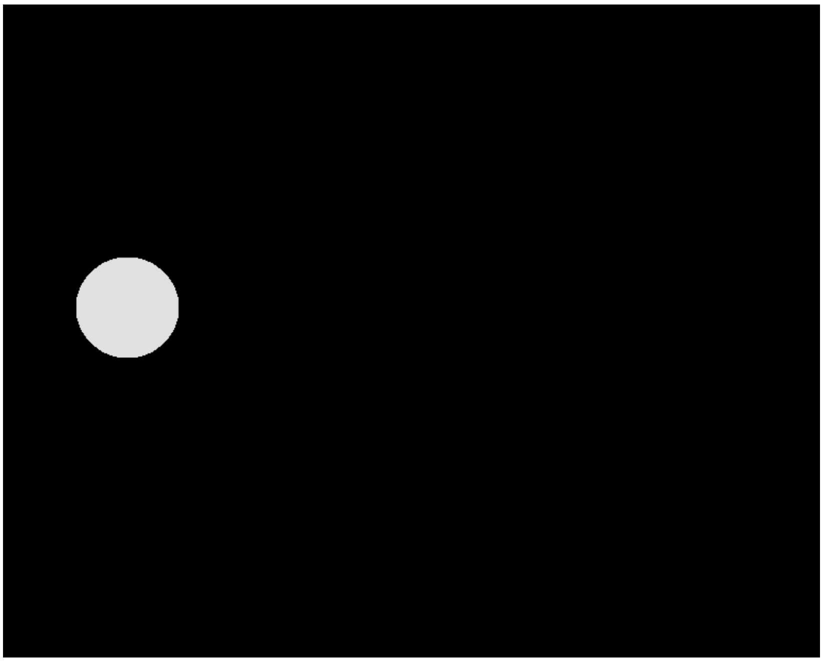

Figure S1 Example of circle (2 cm diameter) for screen training. While screen training, the hens learned to use the screen in combination with the food reward delivery system. Therefore, the circle was shown at each trial at a randomized position.

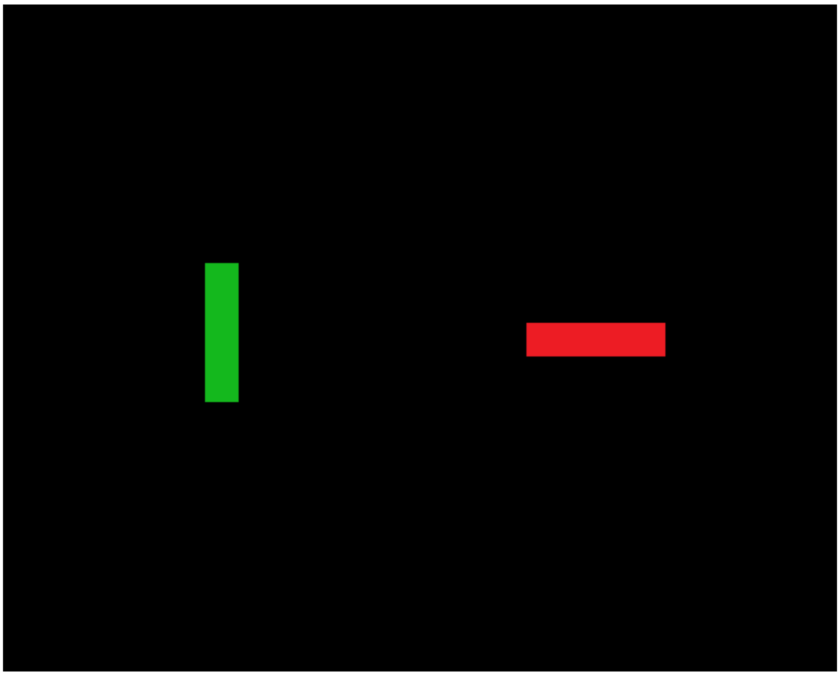

Figure S2 Example of stimuli for discrimination, reversal and extinction learning. Two bars, on green and one red (each high x length: 10 mm x 40 mm) were represented with a randomized orientation and side on a black screen. During discrimination and reversal learning, on color was always rewarded. During extinction none of the colours were rewarded.

Trials of learning phases

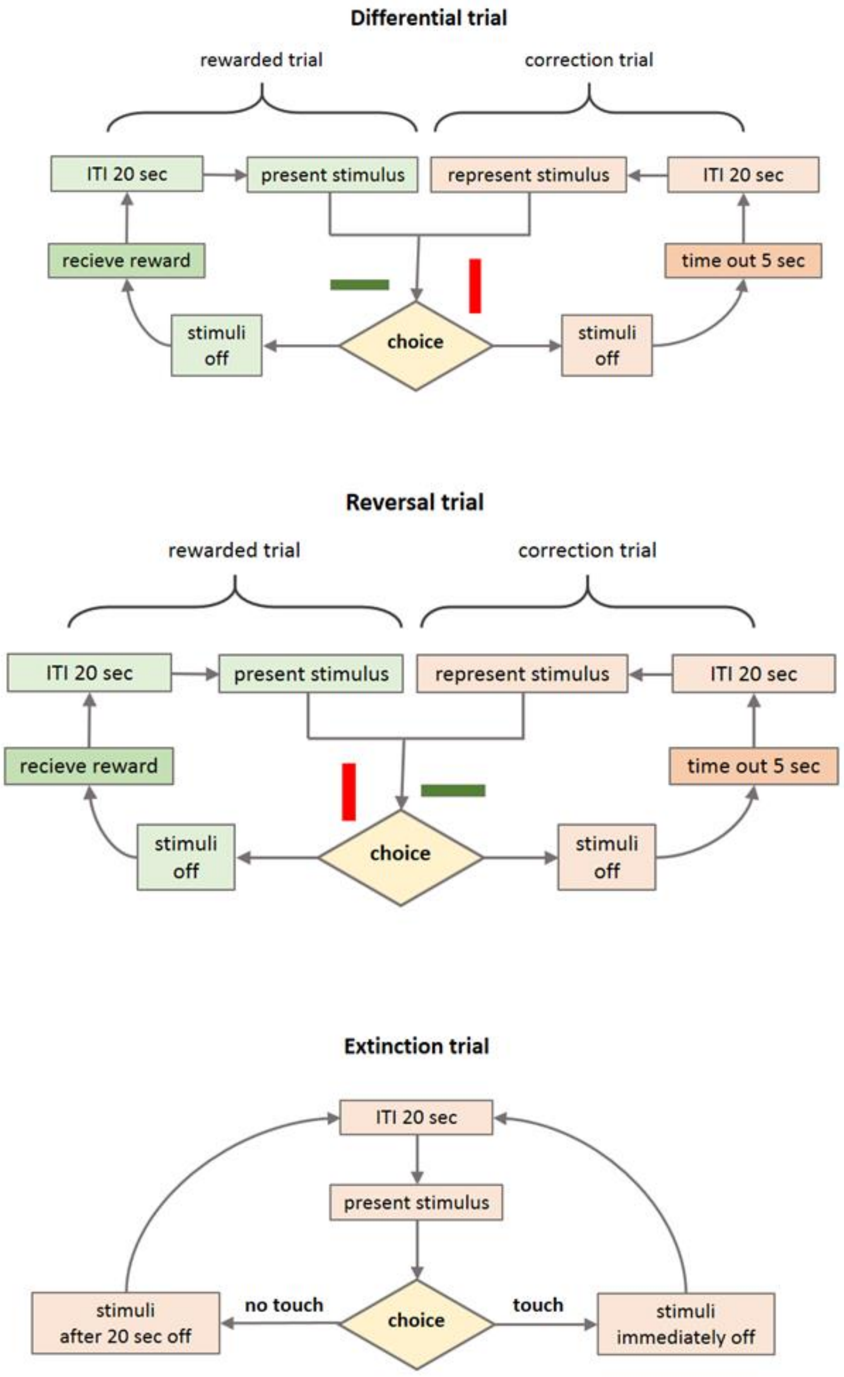

Figure S3 Flowchart overview of trials of different learning phases, adapted after Mar et al. 2013 (Mar et al., 2013). One counted trial is in that case one loop on either side of the flowchart. ITI (intertrial interval) represents the intercomponent time, which is a short break in-between and considered to improve learning performance of animals (Bussey et al., 2008).

References

Bussey, T.J., Padain, T.L., Skillings, E.A., Winters, B.D., Morton, A.J., and Saksida, L.M. (2008). The touchscreen cognitive testing method for rodents: how to get the best out of your rat. *Learning & Memory* 15(7)**,** 516-523.

Mar, A.C., Horner, A.E., Nilsson, S.R.O., Alsiö, J., Kent, B.A., Kim, C.H., et al. (2013). The touchscreen operant platform for assessing executive function in rats and mice. *Nature Protocols* 8**,** 1985. doi: 10.1038/nprot.2013.123.
